# Supplementary material for: ConCISE: Consensus Annotation Propagation of Ion Features in Untargeted Tandem Mass Spectrometry Combining Molecular Networking and In Silico Metabolite Structure Prediction
Source: Metabolites. 2022 Dec 16;12(12):1275. doi: 10.3390/metabo12121275 (PMC9786801; doi:10.3390/metabo12121275)
Supplement: Supplementary file 1 [file metabolites-2068345-supplementary.pdf › metabolites-2068345-supplementary.pdf]

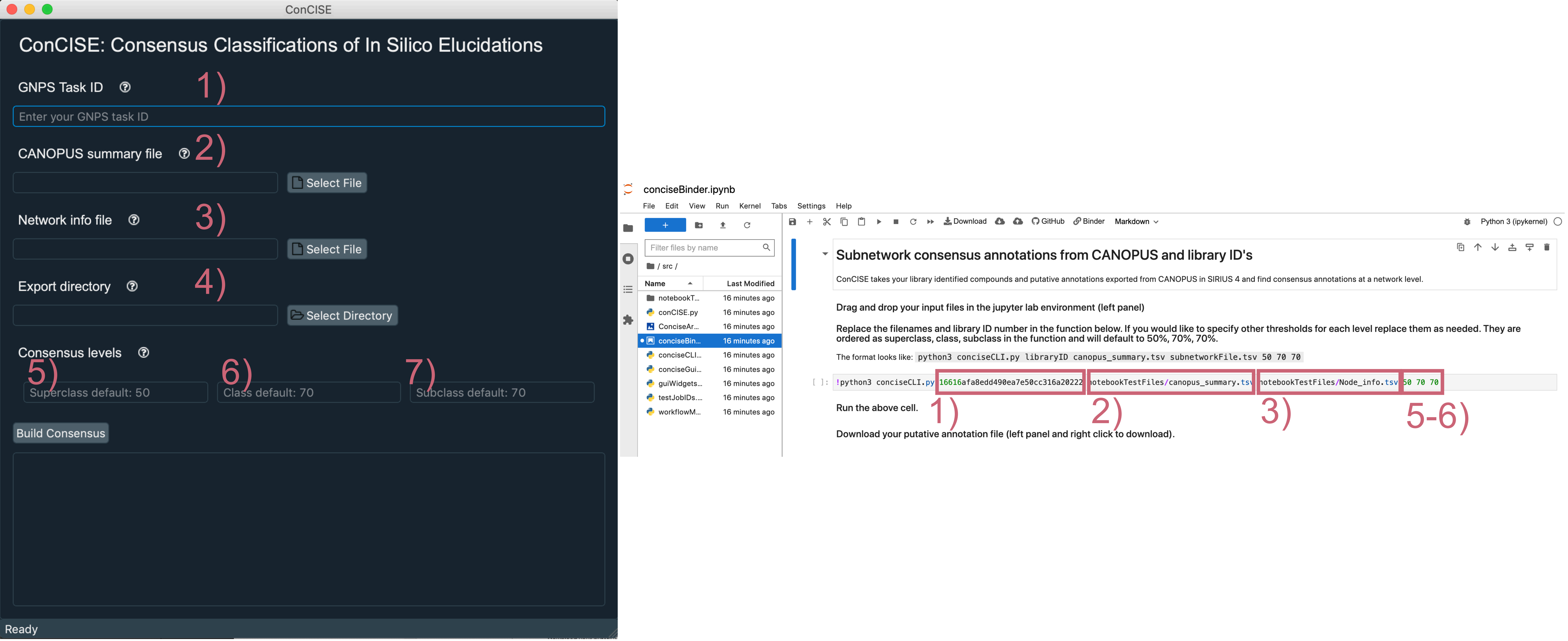
ConCISE
ConCISE: Consensus Classifications of In Silico Elucidations
GNPS Task ID
1)
Enter your GNPS task ID
CANOPUS summary file
2)
Select File
Network info file
3)
Select File
Export directory
4)
Select Directory
Consensus levels
5)
6)
7)
Superclass default: 50
Class default: 70
Subclass default: 70
Build Consensus
Ready
conciseBinder.ipynb
File Edit View Run Kernel Tabs Settings Help
Download GitHub Binder Markdown
Python 3 (ipykernel)
Filter files by name
/ src /
Name
Last Modified
notebookT...
conCISE.py
ConciseAr...
conciseBin...
conciseCLI...
conciseGui...
guiWidgets...
testJobIDs...
workflowM...
16 minutes ago
Subnetwork consensus annotations from CANOPUS and library ID's
ConCISE takes your library identified compounds and putative annotations exported from CANOPUS in SIRIUS 4 and find consensus annotations at a network level.
Drag and drop your input files in the jupyter lab environment (left panel)
Replace the filenames and library ID number in the function below. If you would like to specify other thresholds for each level replace them as needed. They are ordered as superclass, class, subclass in the function and will default to 50%, 70%, 70%.
The format looks like: python3 conciseCLI.py libraryID canopus_summary.tsv subnetworkFile.tsv 50 70 70
!python3 conciseCLI.py 16616afa8edd490ea7e50cc316a20222 notebookTestFiles/canopus_summary.tsv notebookTestFiles/Node_info.tsv 50 70 70
1)
2)
3)
5-6)
Run the above cell.
Download your putative annotation file (left panel and right click to download).
